# Recovery of renal function after glucocorticoid therapy for IgG4-related kidney disease with renal dysfunction

**DOI:** 10.1007/s10157-015-1140-0

**Published:** 2015-07-04

**Authors:** Takako Saeki, Mitsuhiro Kawano, Ichiro Mizushima, Motohisa Yamamoto, Yoko Wada, Yoshifumi Ubara, Hitoshi Nakashima, Tomoyuki Ito, Hajime Yamazaki, Ichiei Narita, Takao Saito

**Affiliations:** Department of Internal Medicine, Nagaoka Red Cross Hospital, Senshu 2-297-1, Nagaoka, 940-2085 Japan; Division of Rheumatology, Department of Internal Medicine, Kanazawa University Hospital, Kanazawa, Japan; Department of Gastroenterology, Rheumatology and Clinical Immunology, Sapporo Medical University School of Medicine, Sapporo, Japan; Division of Clinical Nephrology and Rheumatology, Niigata University Graduate School of Medical and Dental Sciences, Niigata, Japan; Nephrology Center and Okinaka Memorial Institute, Toranomon Hospital, Tokyo, Japan; Division of Nephrology and Rheumatology, Department of Internal Medicine, Faculty of Medicine, Fukuoka University, Fukuoka, Japan; General Medical Research Center, Faculty of Medicine, Fukuoka University, Fukuoka, Japan

**Keywords:** IgG4-related disease, Chronic kidney disease, Tubulointerstitial nephritis, Glucocorticoid, Follow-up

## Abstract

**Background:**

Although renal dysfunction in IgG4-related kidney disease (IgG4-RKD) shows rapid resolution with glucocorticoid therapy, little is known about the appropriate initial glucocorticoid dose for induction therapy or long-term renal outcome.

**Methods:**

We retrospectively examined the differences in recovery of renal function according to the dose of glucocorticoid used for induction therapy and the long-term renal outcome in 43 patients with definite IgG4-RKD (mostly IgG4-tubulointerstitial nephritis), in whom the estimated glomerular filtration rate (eGFR) before glucocorticoid therapy was <60 ml/min.

**Results:**

Most patients were treated with glucocorticoid alone and had been maintained on glucocorticoid. The initial dose of prednisolone employed was ≤0.6 mg/kg/day (mean 0.47) in 27 patients (group L), and >0.6 mg/kg/day (mean 0.81) in 16 patients (group H). In both groups, the pretreatment eGFR was significantly improved at 1 month after the start of glucocorticoid therapy and the degree of improvement showed no significant inter-group difference. Relapse of IgG4-RKD occurred in 16.7 % of the group L patients and 13.3 % of the group H patients (*p* = 0.78). Among 29 patients who were followed up for over 36 months (mean 74 months) and had been maintained on glucocorticoid, none showed progression to end-stage renal disease and there was no significant difference between eGFR at 1 month after treatment and eGFR at the last review.

**Conclusion:**

In glucocorticoid monotherapy for IgG4-RKD, a moderate dose is sufficient for induction, and recovery of renal function can be maintained for a long period on low-dose maintenance, although relapse can occur even in patients receiving maintenance therapy.

## Introduction

IgG4-related disease (IgG4-RD) is a recently recognized fibro-inflammatory condition that can affect multiple organs [1, 2]. It is characterized by fibrosis and a dense lymphoplasmacytic infiltrate including numerous IgG4-positive plasma cells, being usually manifested as tumefactive or hyperplastic lesions in the affected organs subjectively or by radiology. In IgG4-RD, a rapid response to glucocorticoid (GC) therapy is also reported to be characteristic, and clinical symptoms or morphological abnormalities are immediately resolved with GC therapy in most patients, although relapse of the disease is frequent [2]. However, data on organ function in such patients are sparse.

In IgG4-related kidney disease (IgG4-RKD), IgG4-related tubulointerstitial nephritis (IgG4-TIN) is the most dominant feature [3, 4]. In our earlier study of IgG4-RKD, we found that most of the serological and radiological abnormalities and renal dysfunction mostly attributable to IgG4-TIN had improved at 1 month after the start of GC treatment in most patients [3–5]. However, renal function did not recover completely in patients with advanced renal damage. In 22 patients whose estimated glomerular filtration rate (eGFR) before treatment was <60 ml/min, there was a significant improvement of the eGFR at 1 month after the start of treatment (45.0 ± 13.8 ml/min, compared with 34.1 ± 15.8 ml/min before treatment, *p* < 0.01), but recovery of renal function reached a plateau during the initial 1 month of GC treatment in most patients, and thereafter renal atrophy developed in many [5]. In addition, relapse of the IgG4-related lesions occurred in 20 % of the patients with IgG4-RKD treated with GC [5]. These results raised certain questions, such as whether induction therapy with high-dose GC is superior in terms of initial recovery of renal function or relapse of IgG4-RKD in comparison with low-dose GC, or whether patients with pre-existing moderate to severe renal dysfunction will ultimately show progression to end-stage renal disease. Indeed, most of the patients with IgG4-related TIN showed high grades of fibrosis [6], and concerns about progression of fibrosis in IgG4-RD leading to irreversible organ dysfunction have been discussed, although data on organ function are sparse [7]. In the present study, we retrospectively examined differences in recovery of renal function or IgG4-RKD relapse according to the dose of GC used for induction therapy and the long-term renal outcome in a large multi-center cohort of IgG4-RKD patients with renal dysfunction.

## Materials and methods

### Patients

From 16 collaborating institutions in Japan, we retrospectively collected 44 patients diagnosed as having definite IgG4-RKD, in whom eGFR before GC treatment had been less than 60 ml/min. The diagnosis of definite IgG4-RKD was based on the criteria proposed by the Japanese Society of Nephrology [4]. Among the 44 patients, 40 were diagnosed as having IgG4-RKD between January 2006 and April 2013. Four patients were initially diagnosed as having TIN accompanied by primary Sjögren’s syndrome between 1995 and 2001, and the diagnosis was corrected to IgG4-TIN on the basis of histological and serological re-evaluation. Twenty-two of the 44 patients had been included in our earlier study [5]. In one patient with advanced renal failure, hemodialysis became necessary immediately after the start of treatment, and renal function did not recover thereafter. We excluded this patient and enrolled the remaining 43 for the present study. In our earlier study of IgG4-RKD, abnormalities of renal function, serological and imaging features, and extra-renal lesions were all improved at 1 month after the start of GC therapy in most patients, and renal function was maintained at a similar level at 12 months [5]. Therefore, in this study, we also retrospectively examined both treatment and renal function before therapy, 1 month after the start of treatment, 12 months after the start of treatment, at the last review, and at relapse of IgG4-RKD. The study was approved by the review board of Nagaoka Red Cross Hospital and the boards of the collaborating institutions as well as the ethics board of the Japanese Society of Nephrology. All data and samples from patients were collected with their informed, written consent, and the study was conducted in compliance with the principles of the Declaration of Helsinki.

### Definition of remission of IgG4-RKD

Remission was decided on the basis of stabilization or improvement of renal function (as assessed in terms of the serum creatinine concentration or eGFR), improvement of radiological findings, and resolution of extra-renal manifestations [8–10].

### Definition of relapse of IgG4-RKD

Relapse of IgG4-RKD was decided by each attendant physician on the basis of a rapid rise in the serum creatinine level, after careful exclusion of other renal diseases, with re-appearance or worsening of serologic, radiologic (including extra-renal lesions), or histologic features [5, 9]. In IgG4-RD, re-elevation of serological levels alone in the absence of clinical symptoms or abnormal imaging findings was not considered to be relapse because this can also occur when relapse is not evident [8, 9]. Also in our earlier study of IgG4-RKD, the serum IgG4 level remained elevated in 70.8 % of patients in remission [5]. Therefore, re-elevation of the serum level of IgG or IgG4 alone and worsening of urinalysis parameters alone were not considered to be relapse of IgG4-RKD.

### Statistics

Statistical analysis was performed using SPSS version 19 software (IBM SPSS, Chicago, IL, USA). The significance of differences between groups was determined using the paired Student’s *t* test, Mann–Whitney *U* test, or Wilcoxon signed-rank test, and the significance of differences in frequencies was analyzed with Fisher’s exact probability test. Data are presented as mean ± S.D. A probability of *p* < 0.05 was considered to indicate statistical significance.

## Results

### Baseline characteristics

Forty-three patients with definite IgG4-RKD and renal dysfunction who had been treated with GC were enrolled. Their eGFR before GC therapy had been less than 60 ml/min/1.73 m^2^, and none had developed end-stage renal disease within the initial 1 month of therapy. The eGFR before GC therapy was 6.6–59.8 (mean 33.2) ml/min. The results of renal biopsy and the clinical course suggested that the renal dysfunction had been caused by IgG4-RKD (mainly IgG4-TIN). All of the 43 patients were Japanese (38 males and 5 females), and the age at the time of diagnosis of renal disease was 42–85 (mean 66.5) years. The follow-up period after GC treatment was 6–210 months (mean 43 months). Forty-one of the 43 patients (95.3 %) had accompanying IgG4-related extra-renal lesions, and the mean number of extra-renal organs involved was 2.4. In 40 patients, renal needle biopsy was conducted and IgG4-TIN was evident in all of them. In the remaining 3 patients, the diagnosis of IgG4-RKD was based on the characteristic features of the renal parenchyma demonstrated by radiology, in the setting of biopsy-proven IgG4-related lesions in extra-renal organs [4]. Glomerular lesions other than global sclerosis were evident in 7 patients in addition to IgG4-TIN. GC alone was used for induction therapy in all patients, although the dose was decided according to the opinion of each attending physician. The initial dose was continued for 2–4 weeks in all cases, and then tapered gradually to a maintenance dose (5–10 mg/day) in most cases. Forty-one of the patients (95.3 %) had been treated with GC alone during the clinical course. An immunosuppressant (azathioprine or cyclophosphamide) was added for 2 patients on maintenance. At the last review, 40 of the 43 patients had been maintained on low-dose GC.

### Recovery of renal function for induction glucocorticoid therapy

Among the 43 patients, the initial dose of prednisolone employed was ≤0.6 mg/kg/day (0.2–0.6 mg/kg/day, mean 0.47 mg/kg/day) in 27 patients (group L), and >0.6 mg/kg/day (0.61–1.1 mg/kg/day, mean 0.81 mg/kg/day) in 16 patients (group H). The mean follow-up period was significantly longer in group H (85.6 months) than in group L (44.0 months) (*p* < 0.05). There was no significant inter-group difference in the other clinical parameters (Table 1). Glomerular lesions were evident in 4 patients in group L (segmental endocapillary proliferative glomerulonephritis in 1, IgA nephropathy in 1, membranoproliferative glomerulonephritis in 1, and diabetic nephropathy in 1), and 3 patients in group H (membranous nephropathy in 1, Henoch-Schönlein purpura nephritis in 1, and mesangioproliferative glomerulonephritis in 1).

**Table 1:** Baseline clinical features in group L and group H

	Group L (*n* = 27)	Group H (*n* = 16)	*p*
Age (years)	67.3 ± 9.9	65.1 ± 9.6	0.48
Male	24 (88.9)	14 (87.5)	0.89
Hypertension	15 (55.6)	10 (62.5)	0.66
Diabetes mellitus	16 (59.3)	9 (56.3)	0.85
Serum IgG4 level (mg/dl)	1067.7 ± 751.4	1050.1 ± 574.7	0.93
Hypocomplementemia	13 (48.1)	9 (56.3)	0.61
Extra-renal lesion (s)	25 (92.6)	15 (93.8)	0.89
Number of extra-renal lesions	2.6 ± 1.6	2.1 ± 1.3	0.30
Pretreatment eGFR (ml/min/1.73 m^2^)	33.0 ± 16.4	33.6 ± 14.2	0.91
Follow-up periods (months)	44.0 ± 21.0	85.6 ± 63.8	0.027
Initial prednisolone dose (mg/kg daily)	0.47 ± 0.11	0.81 ± 0.16	<0.001

Values for categorical variables are given as number (percentage), values for continuous variables are given as mean ± standard deviation

**Fig. 1:**
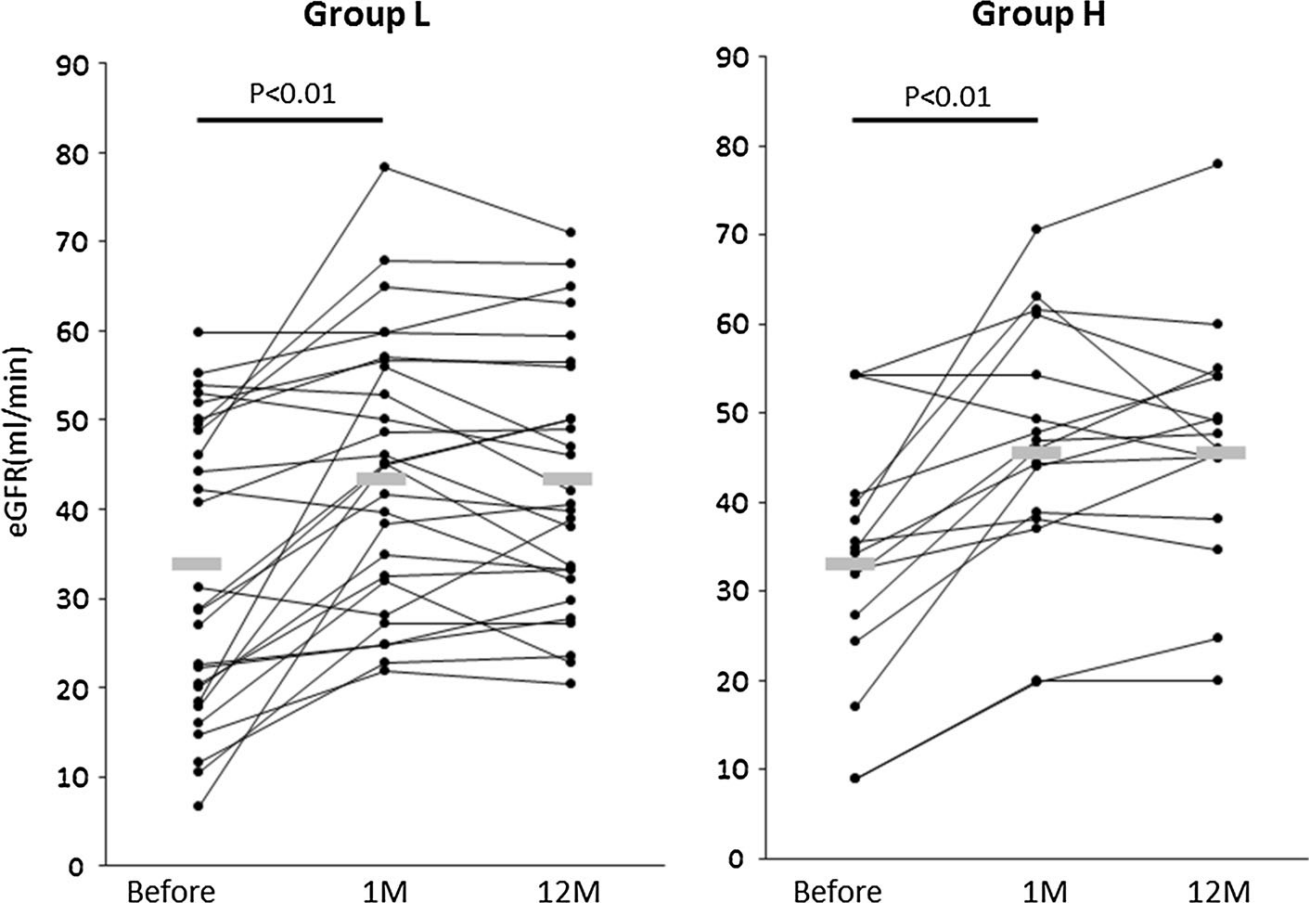
Changes in eGFR after GC therapy. Individual plots of eGFR before GC treatment (Before), at 1 month after the start of treatment (1 M) and at 12 months after the start of treatment (12 M). The mean values at each point are shown adjacent to the *horizontal gray bars*

At 1 month after the start of GC therapy, stabilization or improvement of renal function, improvement of radiological findings, and resolution of extra-renal manifestations were achieved in all 43 patients, i.e., all patients were judged to be in remission. In both groups, the pretreatment eGFR was significantly improved at 1 month after the start of GC therapy [33.0 ± 16.4–44.1 ± 15.4 ml/min in group L (*p* < 0.01) and 33.6 ± 14.2–46.4 ± 14.1 ml/min in group H (*p* < 0.01)], and reached a plateau thereafter (Fig. 1). The degree of improvement during the initial 1 month of GC therapy showed no significant inter-group difference (11.1 ± 11.9 ml/min in group L, 12.8 ± 10.5 ml/min in group H, *p* = 0.63).

### Relapse of IgG4-RKD in patients who had been maintained on glucocorticoid

We examined relapse of IgG4-RKD in 39 patients who had been followed up for over 12 months and had been maintained on GC at the last review. Relapse of IgG4-RKD occurred in 6 patients (4 in group L and 2 in group H) who had been receiving 2.5–10 mg prednisolone daily, and the relapse rate did not differ significantly between group L [4 of 24 (16.7 %)] and group H [2 of 15 in group H (13.3 %)] (*p* = 0.78). All of the 6 relapsed patients were elderly males with several extra-renal lesions, and eGFR before therapy had varied from 6.6 to 54.0 ml/min. Relapse occurred at 22, 18, 12, and 14 months after the start of GC therapy in group L, and at 12 and 16 months after the start of GC therapy in group H; there were no striking inter-group differences in the clinical features of the relapsed patients. In all cases of IgG4-RKD relapse, increasing the dose of GC was effective for resolution. Relapse of extra-renal lesions occurred in 2 patients (type 1 autoimmune pancreatitis and sialadenitis) in group L and 1 patient (lymphadenopathy) in group H.

### Long-term renal outcome of patients who had been maintained on glucocorticoid

Thirty-two of the 43 patients were followed up for over 36 months after GC therapy. Among them, 29 had been maintained with low-dose GC at the last review (Table 2), and these patients were followed up for 37–210 months (mean 74 months). Fifteen of these 29 patients had been included in our earlier study [5] (patients 2, 3, 5, 6, 7, 12, 15, 16, 18, 19, 23, 25, 26, 27, and 29) and 14 were newly enrolled for the present study. The mean maintenance prednisolone dose at the last review was 4.9 mg daily (range 1–10 mg daily, <5 mg daily in 76 %). Among the 29 patients, none showed progression to end-stage renal disease, and there was no significant difference in eGFR at the last review (43.5 ± 14.1 ml/min) in comparison to that at 1 month after the start of treatment (43.5 ± 14.4 ml/min) (*p* = 0.85) (Table 2). Although renal function gradually decreased in some patients, it was not considered to represent relapse of IgG4-RKD by each attendant physician.

**Table 2:** Long-term renal outcome of patients who had been maintained on glucocorticoid

Follow-up				Extra-renal lesions	Renal pathology	Initial PSL (mg/kg/day)	Last PSL (mg/day)	eGFR (ml/min)		
No	Age	Sex	(mo)					Before Tx	1 M after Tx	Last
1	68	M	37	Sa, RF	TIN	0.53	5	15.9	31.9	30.5
2	63	M	38	Sa, Pa, Lu, Ao	TIN	0.31	9	42.2	29.3	41.6
3	75	M	38	Sa	TIN	0.60	5	22.3	24.8	30.9
4	67	M	38	La, Lu, Ly	TIN	0.37	5	11.6	22.7	25.5
5	60	M	39	La, Sa	TIN	0.45	5	31.1	28.1	41.9
6	59	M	46	Sa, Pa, Pro, Ao	TIN	0.80	5	54.2	49.3	35.8
7	61	F	46	Ly, Lu	TIN	0.51	1	18.4	55.9	52.6
8	72	M	46	RF, Ao	TIN	0.60	5	20.4	32.5	36.7
9	63	F	46	La, Sa, Ly	TIN	0.60	8	28.9	45.1	41.5
10	61	M	47	Sa, Lu, Ly	TIN	1.00	10	34.9	61.0	54.4
11	60	M	48	Sa, RF, Pa	TIN	0.39	5	54.2	54.2	41.2
12	68	M	49	Sa, Ly	TIN + IgAGN	0.41	8	28.6	41.6	42.3
13	59	M	51	Ly	TIN	0.62	5	24.4	38.9	59.1
14	85	M	53		TIN	0.36	3.75	53.0	50.0	19.0
15	42	M	55	Sa, La, Pa, Lu	NA	0.51	3	59.8	56.8	58.3
16	75	F	56	Sa, Ly, Lu	TIN + HSPN	0.63	7	17.1	43.9	56.1
17	80	M	58		TIN	0.37	2.5	17.9	45.2	38.7
18	76	M	64	Sa	TIN + MN	0.68	5	8.9	19.7	25.7
19	58	M	66	He, Neu	TIN	0.54	7	45.4	56.8	66.3
20	85	M	67	Pro	TIN	0.26	2.5	14.7	21.8	16.6
21	71	M	71	Sa, Ly, Lu	TIN	0.45	8	46.1	78.4	71.2
22	62	F	74	La, Sa, Pa, RF	TIN	0.6	2	55.2	59.8	70.8
23	83	M	77		TIN + MN	0.85	4	35.5	38.1	30.3
24	84	M	91	Sa, Ly	TIN + MPGN	0.2	2.5	20.0	34.9	30.1
25	68	M	115	Sa	TIN	0.61	5	41.0	47.8	52.0
26	55	M	150	Sa, Pa	TIN	0.8	2	27.3	46.1	49.3
27	60	M	180	Sa, Ly	TIN + MGN	0.73	5	32.5	37.1	49.3
28	45	M	197	Pa	TIN	0.66	2.5	32.0	47.0	49.0
29	61	M	210	Sa, Ly, Pa, Thr	TIN	1.1	5	54.3	61.6	49.6

*mo* month, *PSL* dose of prednisolone, *Sa* sialadenitis, *RF* retroperitoneal fibrosis, *Pa* type 1 autoimmune pancreatitis, *Lu* lung lesion, *Ao* periaortitis, *La* dacryoadenitis, *Ly* lymphadenitis, *Pro* prostatitis, *He* hepatopathy, *Neu* perineuritis, *Thr* thrombocytopenia, *TIN*; IgG4-related tubulointerstitial nephritis, *IgAGN* IgA nephropathy, *HSPN* Henoch-Schönlein purpura nephritis, *MN* membranous nephropathy, *MPGN* membranoproliferative glomerulonephritis, *MGN* mesangioproliferative glomerulonephritis, *NA* not available, *Tx* treatment

### Clinical course of patients in whom maintenance therapy was withdrawn

Maintenance therapy was withdrawn in 3 of the 43 patients. In 2 patients, maintenance GC was stopped according to the opinion of the attending physician at 24 and 36 months after the start of treatment, respectively, and the patients were followed without any maintenance therapy thereafter. At the last review (51 and 4 months after therapy withdrawal), no relapse of IgG4-RD, including IgG4-RKD, had occurred in these patients. In the remaining patient, pretreatment eGFR (40 ml/min) had improved to 60 ml/min at 1 month after the start of GC therapy, but at 2 months the therapy was stopped at the patient’s request because of an adverse event (severe infection). Five months after withdrawal of GC therapy, renal function deteriorated rapidly with re-elevation of the serum IgG4 level. Although relapse of IgG4-RKD was suspected, the patient developed infection several times, and was followed up without any immunosuppressive therapy, including GC, at the patient’s request. At the last review (at 50-month follow-up), the eGFR was 24 ml/min.

## Discussion

Although no formal treatment strategy for IgG4-RD has yet been established, GC is used for first-line therapy in most patients [1, 2], and a moderate dose GC is now recommended for initial treatment of type 1 autoimmune pancreatitis [11, 12]. However, there has been a tendency to use high-dose GC for induction therapy in patients with multi-organ damage or organ failure, because of an underlying unfounded belief that use of a lower dose may predispose to progression of organ damage, or frequent relapse. IgG4-TIN is characterized by a dense lymphoplasmacytic infiltrate and characteristic and high-grade fibrosis [6, 13]. Histologically, IgG4-TIN may appear potentially serious because severe inflammation and marked fibrosis is usually associated with progressive and irreversible organ failure, and in fact renal dysfunction can occur in the absence of therapy [3–5]. In addition, the level of serum IgG4 is highly elevated and multiple organs are affected in most patients with IgG4-TIN [3, 4]. Therefore, high-dose GC had been used as induction therapy for IgG4-TIN, especially at the initial stage after discovery of the disease. However, as prednisolone 0.6 mg/kg/day has been widely employed by Japanese physicians for initial therapy of type 1 autoimmune pancreatitis [11], moderate or relatively small doses of GC have also been tried as induction therapy for IgG4-TIN, even in the absence of any evidence for its effectiveness. In the present study, we found that there was no significant difference in the rate of IgG4-RKD (mostly IgG4-TIN) relapse or recovery of renal function during the initial 1 month of GC therapy between group L (initial dose of prednisolone <0.6 mg/kg/day, mean 0.47 mg/kg/day) and group H (>0.6 mg/kg/day, mean 0.81 mg/kg/day), suggesting that high-dose GC is not necessary, and that a prednisolone dose of 0.5 mg/kg/day is sufficient for induction therapy in IgG4-TIN patients with renal dysfunction. In addition, we found that once renal function had recovered during the first month of GC therapy, it could be maintained for a long period on low-dose GC maintenance therapy alone (mostly less than 5 mg prednisolone daily) in IgG4-TIN.

Although the pathogenesis of IgG4-RD has not been clarified, “storiform fibrosis” has been described as a characteristic feature [14]. This is manifested as a swirling pattern of fibrosclerosing inflammation consisting of inflammatory cells and irregular fibrosis, i.e., fibrosis containing many inflammatory cells but lacking genuine fibrosis [15]. Indeed, in most patients with IgG4-TIN, nests of inflammatory cells surrounded by irregular fibers in the renal interstitium are evident by PAM staining [16], and the pattern of fibrosis is quite different from that in other types of TIN [6]. In addition to this unique inflammatory process in IgG4-RD, the epithelium is relatively well preserved in spite of severe inflammation [15]. Also in IgG4-TIN, severe tubulitis is rare [6]. These features may account for the rapid response of IgG4-TIN to GC therapy and relatively good maintenance of renal function under maintenance therapy, rather than severe inflammation and high-grade fibrosis. Of course, some fibrotic lesions may progress to genuine fibrosis with fewer cellular components, as described for type 1 autoimmune pancreatitis [15], and this can be observed radiologically as gradual progression of renal atrophy. In fact, re-biopsy studies of IgG4-TIN after GC therapy have revealed a mixture of almost normal areas and severely fibrotic areas in the same patient [17]. Also, follow-up CT studies of IgG4-RKD patients after GC have revealed areas showing complete recovery admixed with regions of progressive atrophy in some cases [17, 18].

Although GC is quite an effective treatment for IgG4-TIN, withdrawal may be difficult if GC is being used as monotherapy, although this has not yet been confirmed. Relapse of IgG4-RKD occurred in about 15 % of the present patients, even those being maintained on GC, and progression of renal dysfunction was apparent in 1 of 3 patients after withdrawal of maintenance therapy. Although an increase in the dose of GC is usually effective for treatment of IgG4-RKD relapse [5], and further development to end-stage renal disease is rare, our earlier study indicated that long-term use of GC was associated with various adverse events such as worsening of diabetes mellitus, avascular necrosis of the femoral head and osteoporosis [5], and therefore another treatment strategy should also be considered. Although various immunosuppressive drugs such as azathioprine or mycophenolate mofetil have been tried as steroid-sparing therapy for IgG4-RD, no immunosuppressive drug has been shown to be definitely effective for reduction of the maintenance dose or withdrawal of GC [19, 20]. Recently, B cell depletion therapy (rituximab) for IgG4-RD has been proposed by the North American group, with reduction or withdrawal of maintenance GC, or even monotherapy without GC [21], although data for IgG4-RKD are sparse.

Because this study was retrospective, the most appropriate dose of GC as induction therapy for IgG4-TIN patients remained unclear. Prednisolone at 0.2–0.3 mg/kg/day was effective for recovery of renal function in a few of the present patients, suggesting that a smaller GC dose might also be effective. Further prospective studies will be necessary to determine the most appropriate induction dose of GC for IgG4-TIN.

In addition, glomerular lesions in some cases of IgG4-RKD should be considered when formulating any treatment strategy. In the present study, although patients with glomerular lesions also achieved remission at 1 month after the start of GC treatment, the response in terms of urinalysis parameters after therapy varied, in contrast to the uniform rapid response in terms of renal function, radiology, and serology [5]. In fact, development of end-stage renal disease has been described in patients with IgG4-related membranous glomerulonephritis [22].

In conclusion, our present study has revealed that rapid recovery of renal function can be achieved with a relatively small dose of GC monotherapy in most IgG4-RKD patients, and that recovery of renal function can be maintained for a long period on low-dose GC maintenance in patients for whom induction GC therapy has been successful, although relapse can occur even in patients receiving maintenance therapy. These observations should be informative for establishment of a useful treatment strategy for IgG4-RD and also for considering its pathogenesis.
